# Potential harm from universal school-based mental health interventions: Candidate mechanisms and future directions

**DOI:** 10.1016/j.copsyc.2025.102196

**Published:** 2025-10-15

**Authors:** Lucy Foulkes, Carolina Guzman Holst, Jack L. Andrews

**Affiliations:** 1 Department of Experimental Psychology, University of Oxford, Life and Mind Building, South Parks Road, Oxford, OX1 3PS, UK

## Abstract

Universal school-based mental health interventions involve lessons delivered to whole classes of young people irrespective of need, with the overall aim of improving mental health literacy, preventing mental health problems and/or reducing those that have already started. A number of high-quality trials show that universal interventions can have a range of negative effects, with participants in the intervention group experiencing worsening mental health or other negative outcomes. In this review, we summarise what we know so far about these negative effects, which we refer to as ‘potential harm’. Two important questions remain poorly understood. First, the mechanisms driving potential harm are unknown, including whether negative effects are driven by reporting phenomena, the content of the intervention itself, or both. Second, individual differences in susceptibility to these effects is unclear. In the second half of the paper, we explore whether further universal trials should be run and argue that, if they are, the crucial issue of negative effects must be considered at all stages. In particular, we recommend that trials be designed in such a way to test mechanisms and individual differences in response. Information can then be provided to schools and policymakers about why young people might respond in different ways to an intervention, and how to support vulnerable students. Ultimately, this will lead to more effective and less harmful interventions for everyone.

## Introduction

Schools are popular settings in which to deliver mental health interventions. These interventions typically involve lessons delivered to groups, either to select young people deemed to be at risk (*targeted* or *indicated interventions*) or to whole classes regardless of need (*universal interventions*). In this review, we focus on universal interventions. These lessons generally involve a combination of general psychoeducation and techniques adapted from specific therapies such as cognitive behavioural therapy (CBT) or mindfulness, and the overall aim is to improve mental health literacy, prevent mental health problems and/or reduce those that have already started.

Meta-analyses indicate that, on average, universal school mental health interventions do improve mental health problems, albeit with small effect sizes and only over the short term [1–3]. However, the majority of evaluation studies included in these meta-analyses are of low quality, potentially biasing overall estimates [1,2,4]. Recent, well-powered studies with large sample sizes show less promising results [4]. Specifically, large trials have found a combination of null and negative effects in universal interventions focused on CBT [5,6], mindfulness [7], dialectical behavioural therapy [8] and general mental health awareness [9].

A recent scoping review evaluated school-based group mental health interventions based on CBT and/or mindfulness and found 8.93% interventions (10 out of 112) reported at least one negative outcome; of those rated as high quality (i.e. those with low risk of bias), 33.33% (five out of 15) reported at least one negative outcome [4]. All negative effects in this scoping review were found in universal, not targeted, interventions. Together, these findings indicate that it is not a specific therapeutic modality or curriculum content that can lead to negative effects. Instead, findings suggest that it might be the universal approach and delivery that can be problematic [10].

The specifics of negative effects vary widely across trials. Negative effects have been reported across a range of self-report measures including a worsening of symptoms of anxiety, depression, hyperactivity/inattention difficulties, obsessive compulsive disorder and panic, and a decrease in parental relationship quality, prosocial behaviour and wellbeing [4,8,9]. In some studies, negative effects were found across all participants, and in others they were found for subgroups of participants, including individuals with elevated levels of mental health problems, male participants, younger participants and those eligible for free school meals [4]. Given the heterogeneity in the literature to date, it is not yet possible to draw confident conclusions about which interventions cause specific negative effects to which specific subgroups.

In this review, we summarise the literature on negative effects in universal school-based mental health interventions and consider next steps. Specifically, ‘negative effects’ refers here to either an increase in an undesirable outcome (e.g. depressive symptoms) or a decrease in a desirable outcome (e.g. prosocial behaviour) in the intervention group; we are not reviewing the presence of discrete adverse events (e.g. hospitalisation) given how rarely they are reported in school mental health intervention trials [11]. Given that negative effect sizes in this literature are typically small, we are cautious about describing negative effects as evidence of definitive harm, and instead refer to them as *potential harm* [4,11]. We review candidate mechanism for these effects, and then highlight that researchers in this field are now faced with a dilemma: whether or not to conduct further universal trials that might be beneficial but might lead to potential harm [10,12]. We end the paper by recommending a number of future research directions, both analytical approaches using existing datasets and, if future trials are run, practical considerations to minimise potential harm, examine mechanism and understand individual differences.

### Candidate mechanisms

It is unclear why negative effects occur in universal interventions, but a number of authors have proposed various possible mechanisms [4]. Given the heterogeneity of negative effects found to date, there are likely many different mechanisms involved depending on the specific intervention, outcome and/or affected subgroup, and thus multiple explanations should be considered in parallel. Before we describe the possible mechanisms in detail, it is important to acknowledge that the presence of negative effects does not necessarily mean that the *content* of the intervention itself is causing the negative outcome. We clarify this in more detail below.

The first possibility is that symptoms or other problematic outcomes are not actually increasing, but rather the content of the intervention has led young people to complete the self-report items more accurately [13–15]. Specifically, the suggestion is that the psychoeducation component of the intervention has reduced stigma and/or increased mental health literacy, leading participants to more accurately and honestly report their experiences. For this explanation, the authors rely on the assumption that ‘more accurate’ post-intervention reporting inevitably means more *negative* scores – although, in reality, more accurate reporting might also mean more positive scores. This explanation is thus the most optimistic interpretation of negative effects, because it implies that negative effects are merely a reporting artefact. Given that a common goal of universal interventions is to improve mental health literacy, this possibility should always be considered. However, other mechanisms that indicate a genuine negative effect should be considered in parallel.

One such explanation is that universal interventions increase young people’s tendency to notice and focus on negative emotions and other symptoms, and that this attention and awareness can lead to negative effects in several ways [7,16–18]. First, increased awareness may lead to rumination, where young people passively and repeatedly think about their emotions either alone (rumination) or with a friend (co-rumination) [13,19,20]; this process has been shown to increase symptoms [21,22]. Second, it may be that universal interventions increase awareness of negative emotions but do not provide enough effective guidance on how to manage or reduce them, and this increased awareness in the absence of a resolution increases distress or other symptoms [18,23]. Some authors have proposed that this may be particularly likely to occur in young people already experiencing high levels of symptoms [18]. Indeed, in any given classroom, many individuals are already symptomatic; thus delivering an intervention designed to be preventative to individuals who are already unwell may be harmful [10,24]. Relatedly, when seeking to explain negative effects on prosocial behaviour and parental relationship quality, some authors have argued that universal interventions encourage a self-centred focus on one’s own emotional experiences, which then comes at a cost to the young person’s relationships with their peers and parents [8,14].

The social context of universal interventions may also cause or contribute to negative effects [25]. In universal interventions, young people are asked to learn about and reflect on their mental health in a classroom full of their peers, and these lessons are typically mandatory, because they take place during the school day [11]. However, many young people do not feel safe at school [26] and are self-conscious and concerned about social evaluation by their peers [27]. Being required to participate in mental health lessons in front of peers during this developmental stage might increase distress or other symptoms [25,28]. Other authors have proposed that universal interventions may lead to problematic social comparisons, where young people with elevated symptoms and/or who are not benefiting from the intervention compare themselves unfavourably to their peers who appear to be coping better than them, and that it is this social comparison which increases distress or other symptoms [14]. To date, however, all these suggestions are based on theory and qualitative data; they have not been evaluated as a possible mechanism for negative effects.

### The dilemma in the field

Much more work is needed to better understand which individuals are at risk of potential harm from universal school-based mental health interventions, and the mechanisms driving such effects. However, given that there is now clear evidence that negative effects exist, this presents the field with an ethical dilemma. On the one hand, we could stop running universal intervention trials and focus on one-to-one or targeted approaches [10]. This would remove the risk of potential harm but leave the field with a poor understanding of the mechanisms driving negative effects or who might be vulnerable. On the other hand, researchers could continue running universal interventions, whilst collecting important mechanistic data and measuring individual differences variables that will allow us to understand why some interventions work well and others do not or lead to potential harm. Importantly, even though positive outcomes can never be guaranteed, trials should only be run when there is reason to expect positive effects based on prior studies of a similar nature [29]. Indeed, it has been recommended that if a trial is high risk/low benefit, or not likely to lead to useful positive effects that are sustained over time [30], then it is not appropriate for researchers to run such a trial [29]. Instead, time should be spent on other approaches to understand mechanisms in the absence of new trials (e.g. with existing data or simulation approaches), and exploring alternative intervention options [10,29].

What complicates the situation further is the limitations of analysing existing trial data to answer crucial questions of mechanisms and individual differences. Most previous trials do not have the necessary data to run these types of analyses; for example, trials may lack the measures, temporal sequencing and power required to establish a mediation pathway with confidence. Even if the measures are available to run mediation analysis, trials are typically powered only for testing main effects, so any secondary analysis to explore mechanisms would likely be underpowered and unreliable. Similarly, trajectory modelling (where participants with similar symptom pathways across an intervention are grouped together) is a valuable means of assessing individual differences in intervention response, but this analysis requires data from at least three time points and ideally a sample size of over 500 individuals [31,32]. Most trials to date do not meet these criteria, particularly those occurring in middle- or low-income countries. In sum, relying only on existing trial data is unlikely to drive meaningful shifts in our understanding of why negative effects happen or which young people are most at risk.

### Future research recommendations

Given the ongoing debate, it is likely that trials evaluating universal school-based mental health interventions will continue. Therefore, in this section, we recommend a number of methodological considerations when planning future universal trials. First, such trials should recruit larger, more diverse samples. This would enable researchers to conduct trajectory analysis, to examine whether different groups respond to the intervention in different ways, and whether age, gender, cultural or ethnic background, pre-existing difficulties, or other variables of interest predict membership into these different groups. This would allow researchers to understand which individuals are most likely to experience negative outcomes from an intervention. To do this effectively, trial designs should include at least two time points after the intervention to understand long-term effects, as well as at least two time points *prior* to the intervention to capture the context, state and variability of mental health symptoms before an intervention takes place. This would help contextualise an individual’s mental health symptoms before the intervention, thus helping to distinguish true intervention effects from any natural symptom fluctuation over time that may occur regardless of the intervention.

Second, as previously mentioned, new universal trials should be adequately powered to analyse secondary outcomes (variables that are not the primary focus of the study’s hypotheses), including moderators and mediators of intervention effects. Third, researchers should consider research designs that enable them to investigate which specific components of an intervention (e.g. CBT problem-solving techniques, emotion regulation skills, peer engagement, mindfulness components, psychoeducation) impact which outcomes. This work has been done in the context of one-to-one psychological therapy and would be valuable for establishing whether specific components lead to positive, negative or null effects [33,34]. Fourth, new universal intervention trials should collect qualitative data to understand in detail why and how some young people find these interventions difficult or unpleasant, or equally, why and how others find them helpful [20]. Fifth, new trials could include self-report measures designed to assess possible negative experiences from an intervention, such as the Scale for Measuring Positive and Negative Experiences of Psychotherapy (PNEP), adapted for the school mental health intervention context with adequate psychometric evaluation [35]. These findings, along with any negative effects from primary or secondary outcomes, qualitative data or specific adverse events, should be openly and systematically reported in all publications [11].

Finally, we recommend that, during informed consent processes, parents and young people themselves should be told about potential negative effects, giving them the option to opt-out if desired (opt-out is typically not available in universal interventions [11]); this might skew the resulting sample of participants but would at least offer a route to reduce potential harm for the most vulnerable young people. Together, these steps will allow researchers to better understand which young people are most likely to experience negative effects from universal school interventions and why, ultimately allowing us to tailor future school interventions to minimise potential harm.

A final point to note is that most existing trials have been conducted in high-income countries, leaving a significant gap in evidence from low- and middle-income countries (LMICs), where priorities and resources differ and universal approaches might be the only affordable approach. Interventions that focus on strengthening protective factors for mental health, such as improving school climate or general healthy behaviours, may be particularly valuable in LMICs, where systemic challenges and contextual stressors play a large role in shaping young people’s mental health [36,37]. For example, in Bihar, India – a low-resource, low-income setting – a multicomponent, universal school health-promotion intervention (SEHER), delivered by lay counsellors in a high-quality trial, resulted in substantial reductions in depressive symptoms among a number of other outcomes, including improvements in school climate and reductions in bullying, violence, and negative gender attitudes, with effects being maintained across two years of follow-up [38,39]. While more research is urgently needed in these settings, we recommend the same principles of considering and measuring negative effects (including those in primary and secondary outcomes as well as any negative experiences identified via specific psychometrically sound self-report measures) [11].

## Conclusion

Over the past decade, enthusiasm for universal school-based mental health interventions has led to a large number of research studies. However, recent, high-quality trials have revealed that universal interventions can have a range of negative effects, with participants in the intervention group experiencing worsening mental health or other negative outcomes [4,8,9]. If researchers continue running trials of universal interventions, this crucial ethical issue must be considered at all stages of the research [11]. In particular, we recommend that new trials be designed in such a way that we can address two unanswered questions: what factors cause interventions to have negative (or positive) outcomes and which young people are more likely to benefit or are most at risk of potential harm. With this knowledge, the field can move towards designing school-based mental health interventions that take into account individual differences and vulnerabilities, leading to a more effective and less harmful intervention for everyone.
